# Aberrant Expression of *BTLA*, *CD160*, *SPN*, *TIM-3*, *VISTA* and *TIGIT* in Chronic Lymphocytic Leukemia and Psoriasis Patients Compared to Healthy Volunteers

**DOI:** 10.3390/cancers17132116

**Published:** 2025-06-24

**Authors:** Katarzyna Skórka, Anita Wdowiak-Filip, Grażyna Stasiak, Joanna Bartosińska, Dorota Krasowska, Krzysztof Giannopoulos

**Affiliations:** 1 Department of Experimental Hematooncology, Medical University of Lublin, 20-093 Lublin, Poland; grazyna.stasiak@umlub.pl (G.S.); krzysztof.giannopoulos@umlub.pl (K.G.); 2 Department of Cosmetology and Aesthetic Medicine, Medical University of Lublin, 20-093 Lublin, Poland; anita.wdowiak-filip@umlub.pl (A.W.-F.); joanna.bartosinska@umlub.pl (J.B.); 3 Department of Dermatology, Venereology and Pediatric Dermatology, Medical University of Lublin, 20-081 Lublin, Poland; dorota.krasowska@umlub.pl

**Keywords:** chronic lymphocytic leukemia (CLL), psoriasis (Ps), immunoregulatory check points, *BTLA*, *CD160*, *CD43(SPN)*, *TIGIT*, *TIM3*, *VISTA*

## Abstract

Background: Currently, much attention is focused on the interactions between the leukemic and psoriatic cells showing immunosuppressive activity within the microenvironment. Methods: Our study assessed a collective mRNA expression pattern of crucial immuno-regulatory genes: *BTLA*, *CD160*, *SPN*, *TIM-3*, *VISTA*, *TIGIT*, by qRT-PCR, and performed a comparison in two different diseases, chronic lymphocytic leukemia (CLL) and psoriasis (Ps), referring to clinical characteristics. Results: In Ps, all the studied gene expressions, except *TIM-3*, were higher than in HVs and all the studied gene expressions, except *VISTA*, were lower than in CLL. However, the expression of *TIM-3*, a checkpoint inhibitor, was higher in 0 stage of CLL and was lower in advanced stages of the disease, suggesting its possible diagnostic value. Expression of *VISTA* was higher in Ps than in HVs, as well as in CLL. It is noteworthy that *BTLA*, *CD160* and *SPN* were overexpressed in CLL and Ps compared to HVs, suggesting its involvement in immune suppression in both diseases. Conclusions: Significant correlations between gene expressions of *SPN* and *BTLA*, *SPN* and *TIGIT*, *CD160* and *TIM-3*, were observed, indicating a potential shared regulatory mechanism for immune responses which suggests their bidirectional regulatory role on the functioning of immune system cells, depending on the context of inflammatory or neoplastic conditions.

## Simple Summary

Currently, much attention is paid to the interactions between the leukemic and psoriatic cells showing immunosuppressive activity within the microenvironment, and, thereby, we aimed to characterize a collective mRNA expression pattern of crucial immuno-regulatory genes: *BTLA*, *CD160*, *SPN*, *TIM-3*, *VISTA* and *TIGIT* and perform a comparison in chronic lymphocytic leukemia (CLL) and psoriasis (Ps). Although Ps is characterized by excessive immune activation, and CLL is marked by immune suppression and escape, interestingly, we observed some overlapping patterns of immune checkpoint dysregulation. *BTLA*, *CD160*, *SPN* were overexpressed in CLL and Ps compared to HVs, suggesting its involvement in immune suppression in these diseases. Significant correlations between *SPN* and *BTLA*, *SPN* and *TIGIT*, *CD160* and *TIM-3* were shown, suggesting a potential shared regulatory mechanism for immune responses in both diseases which indicates their bidirectional regulatory role in the functioning of immune system cells, depending on the context of inflammatory or neoplastic conditions.

## 1. Introduction

Of particular importance in the abnormal stimulation of the immune system are phenomena taking place in the tissues that constitute the external protective barriers of the human body, mainly in the skin. At the root of the inadequate immune response causing inflammation in psoriasis (Ps), as well as chronic lymphocytic leukemia (CLL), are disorders of the immune system, which are a consequence of the interplay between genetic conditions and the impact of environmental factors. Factors that contribute to cytokine production and T-lymphocyte stimulation by interacting with cells of the innate immune system are also involved in the pathogenesis of these diseases. Ongoing pathological processes reduce the tightness of the skin barrier, from which they facilitate the penetration of antigens and pathogens, which promotes further stimulation of the immune system [1]. CLL and Ps represent two distinct diseases, with CLL as a hematologic malignancy characterized by immune suppression, and Ps as an autoimmune disease with an excessive immune activation. Despite their different origin, both conditions share some common features of immune dysregulation.

In hematological malignancies, including CLL, immune deregulations are very common. CLL is a highly frequent leukemia in adults living in Western countries and is characterized by a very heterogeneous clinical course. In CLL patients, both the symptoms of immunosuppression, manifesting by frequent infections, and the occurrence of autoimmunity leading to autoimmune cytopenias are due to qualitative, as well as quantitative, abnormalities of the immune cells. Very important are abnormal interactions between the leukemic cell clone and cells showing immunosuppressive activity within the microenvironment. Crucial impact in immunosuppression in terms of the escape of the immune system from immune surveillance is provided by the programmed death receptor 1 and its ligand (PD-1/PDL-1) signaling pathway. Important immunosuppressive cells in CLL patients include T regulatory (Treg) and B regulatory (Breg) lymphocytes, as well as myeloid-derived suppressor cells (MDSCs). The most significant disruption of the B-cell response is the commonly observed hypogammaglobulinemia. Immunosuppression is clinically manifested by an increased frequency of infections, as well as secondary cancers [2,3,4].

Ps is a chronic T-cell-mediated inflammatory disease of the skin and joints that is estimated to affect 0.1% of the population in East Asia and 1.5% in Western Europe, with an increasing incidence in developing countries [5]. In Ps, activated dendritic cells produce tumor necrosis factor (TNF) α, TNF-β, interleukin 2 (IL-2), interleukin 3 (IL-3), interleukin 22 (IL-22) and interleukin 26 (IL-26), contributing to the differentiation of T cells into Th1 and Th17. Components of the IL-23/Th17 axis interact with skin epithelial cells to initiate and sustain the inflammatory process in both diseases [1,6]_._ At least nine regions of Ps susceptibility risk have been identified based on genome-wide linkage analysis. Based on the genome-wide association studies (GWASs), several conclusions can be drawn about genetic factors in Ps. Most of the genes involved also have immune functions, highlighting the importance of the innate, as well as acquired, immune response [7]. In contrast, relatively few genes that encode skin-specific proteins have been linked to Ps. Related genes encode proteins that have roles in specific immune pathways and signaling pathways, specifically involving tumor necrosis factor-α (TNF-α), nuclear factor κ-b (NF-κB), interferons and IL23/Th17 interleukins. In addition, endoplasmic reticulum aminopeptidase 1 (ERAP1), which encodes an aminopeptidase involved in MHC class I antigen processing, interacts synergistically with the HLA-Cw6 risk allele, providing another argument for the role of major histocompatibility (MHC) antigen and its presentation by human leukocyte antigen (HLA) C in Ps pathogenesis [8]. It is worth noting that the initiators of the development of a disease such as Ps are environmental factors in genetically predisposed individuals. These include infections, hormonal factors, stress, certain medications, alcohol consumption, smoking, and obesity, among others.

Many studies have confirmed the close link between cancers and autoimmune diseases, but the detailed mechanisms and pathophysiology have not been elucidated, which is an obstacle to disease prevention and treatment [9,10]. It has been suggested that patients with Ps are at increased risk of cancer, since many risk factors for cancer development, including smoking and alcohol consumption, are associated with Ps. It has been proven that patients with Ps have an increased risk of developing both Hodgkin’s lymphoma and non-Hodgkin lymphoma [9,10]. This increase may be partly explained by the increased risk of cutaneous T-cell lymphoma (CTCL) in patients with Ps [11].

Moreover, patients with CLL often have weakened immune systems, which can increase the risk of developing various cancers, including skin cancers like basal cell carcinoma, squamous cell carcinoma, melanoma and Merkel cell carcinoma [12,13,14]. In addition, some patients may be treated with therapies that can affect the skin or change its sensitivity to UV radiation, which can also contribute to an increased risk of skin cancers. It is important for patients with CLL to see their dermatologist regularly and have a dermatoscopy check-up so that early detection of any skin changes can lead to more effective treatment [12,13,15]. CLL might be also accompanied by autoimmune phenomena such as autoimmune hemolytic anemia or immune thrombocytopenia, suggesting that elements of autoimmune imbalance exist even in the context of malignancy [16].

Currently, a lot of attention is paid to the interactions between the leukemic, as well as psoriatic, cells, and cells showing immunosuppressive activity within the microenvironment, especially immuno-regulatory markers present there. B and T lymphocyte attenuator (BTLA) is an inhibitory receptor that plays a significant role in limiting inflammatory response. It is crucial to inhibit homeostatic expansion and activation of both the lymph node and skin of T cells. BTLA increases the expression of T regulatory cells, and it provides a negative regulatory effect on Th17, as well as Th1 cell immune responses [14]. *CD160* is a gene encoding a protein of the same name, which belongs to the family of immunoglobulin-like receptors that activate natural killer (NK) cells. As with other NK receptors, CD160 binds classical and non-classical major histocompatibility complex (MHC) class I antigens, including HLA-C and HLA-G. Binding of CD160 to HLA-C is a mechanism triggering NK cell-mediated cytotoxicity and cytokine production. CD160 acts as a receptor coactivator for CD4+ CD16-T cells isolated from inflammatory lesions in the skin [17]. Sialophorin (SPN), otherwise known as superficial protein CD43, is encoded by *SPN* gene that is known to be expressed on the surface of T lymphocytes, natural killer (NK) cells, monocytes, granulocytes, and B lymphocytes, and has been shown to be an important regulator of immune system cell function. CD43 is involved in the regulation of such cellular processes as cell proliferation and adhesion [18,19]. Tim-3 plays an important role in immune tolerance through negative regulation of pro-inflammatory signaling, is constitutively expressed on human NK cells, and can be induced upon activation, ultimately delivering inhibitory signals through crosslinking. In chronic cases such as advanced melanoma, lung adenocarcinoma and chronic hepatitis B, it has been shown that prolonged Tim-3 expression can lead to a depleted/functional NK cell phenotype, which can be prevented by Tim-3 blockade [20]. VISTA is expressed on both antigen-presenting cells (APCs) and T cells, providing inhibiting T-cell activation signals through both extrinsic and intrinsic mechanisms. VISTA acts as a ligand when it is expressed on APCs and engages a putative inhibitory receptor on the T cell that inhibits T cell proliferation as well as cytokine production. However, VISTA undergoing expression on T cells may engage the putative inhibitory receptor on T cells through T cell interaction or act as a self-signaling receptor. Both mechanisms contribute to T cell suppression [21]. TIGIT is another inhibitory receptor. It is expressed on T, NK and NKT (Natural Killer-T) cells, and is involved in suppressing the immune response in various clinical conditions, including cancer. TIGIT shares functional and structural similarities with CTLA-4 and PD-1, respectively. The cytoplasmic tail contains a phosphorylation motif that is similar to the immunoglobulin tyrosine tail (ITT) and an ITIM domain through which TIGIT recruits SHIP1 phosphatase and inhibits activation of the NF-κB, phosphoinositide 3-kinase (PI3K) and mitogen-activated protein kinase (MAPK) pathways, which is similar to that described for PD-1. Moreover, TIGIT binds to CD226/DNAM-1 (DNAX Accessory Molecule-1), so that they compete to bind to the same set of ligands, leading to entry into opposite signaling pathways. The two molecules partially share an expression pattern; however, CD226 is more widely expressed on immune cells, whereas TIGIT is absent on virgin T cells, but is expressed on activated and memory T cells, Treg cells, as well as on NK and NKT cells [22].

Therefore, the current study aimed to present a collective expression pattern of crucial immuno-regulatory genes including *BTLA*, *CD160*, *SPN*, *TIM-3*, *VISTA* and *TIGIT* on the mRNA level, as well as perform a comparison with two different diseases, CLL and Ps, referring to recognized prognostic markers as well as clinical characteristics. Most of the genes that we studied are proved to show immune-regulatory function referring to T subpopulations. There are limited data on their pattern expression on B cells especially at the mRNA level.

## 2. Materials and Methods

The material was obtained from 85 psoriatic patients and 74 CLL patients, as well as 15 healthy volunteers (HVs).

The cohort of patients with Ps were hospitalized at the Department of Dermatology, Venereology, and Pediatric Dermatology at the Medical University of Lublin in Poland. They had not been administered any anti-psoriatic treatment for at least 6 months before being recruited into the study. This cohort included 71 males and 14 females, with the median age of 47. A total of 35.3% patients had concomitant psoriatic arthritis. The severity of Ps was assessed with the use of the Ps Area and Severity Index (PASI). The median value of the PASI was 12.1, with the range from 1 to 49.4.

The cohort of CLL patients involved 46 males and 28 females with the median age of 66. Patients were previously untreated and newly diagnosed at four Polish institutions, including the Department of Hematology, St. John’s Cancer Centre, as well as the Department of Hematooncology and Bone Marrow Transplantation in Lublin.

The clinical characteristics of the CLL and Ps patients are shown in Table 1 and (particularly) Table 2 (Table 1 and Table 2).

### 2.1. Cell Isolation

Peripheral blood mononuclear cells (PBMCs) were obtained from individuals with Ps and CLL, as well as HVs. PBMCs were isolated using Ficoll density gradient centrifugation (Biochrom AG, Berlin, Germany). After isolation, PBMCs were cryopreserved at −80 °C until further analysis. Cell viability consistently obtained >95%, as determined by Trypan blue staining. The number of viable cells were quantified in a Neubauer chamber (Zeiss, Oberkochen, Germany).

### 2.2. RNA Isolation and Reverse Transcription Reaction (RT)

RNA was isolated using QIAamp RNA Blood Mini Kit (Qiagen, Venlo, The Netherlands) according to the manufacturer’s protocols. The quality and quantity of the obtained RNA were quantified spectrophotometrically with the measurement of OD 260/280, using a BioSpec-nano spectrophotometer (Shimadzu, Kyoto, Japan). The RNA samples were stored at −80 °C until further analysis. RT was performed using a QuantiTect Reverse Transcription Kit (Qiagen, Venlo, The Netherlands). For each sample, 1 µg of mRNA was reverse transcribed into 20 μL of cDNA.

### 2.3. Assessment of BTLA, CD160, SPN, TIM-3, VISTA and TIGIT mRNA Expression Using Real-Time Reverse Transcription–Polymerase Chain Reaction (qRT-PCR)

cDNA was used in a qRT-PCR to measure the mRNA expression of *BTLA* (Hs00699198_m1), *CD160* (Hs01073987_m1), *SPN* (CD43; Hs01872322_s1), *TIM-3* (*HAVCR2*; Hs00262170_m1), *VISTA* (*C10orf54*; Hs00735289_m1) and *TIGIT* (Hs00545087_m1) with the use of the TaqMan Gene Expression Assay (Applied Biosystems, Foster City, CA, USA), as well as the 7500 Fast Dx Real-Time PCR Instrument (Applied Biosystems, Foster City, CA, USA), according to the manufacturer’s instructions. The glyceraldehyde-3-phosphate dehydrogenase (*GAPDH*) was used as a reference gene. Moreover, negative control was performed, in which 1 µL of distilled water was added. The thermocycling program was set for 40 cycles of 15 s at 95 °C and 1 min at 60 °C. The *BTLA*, *CD160*, *SPN*, *TIM-3*, *VISTA* and *TIGIT* mRNA expression were calculated using ΔΔ*C_t_* methodology ($2^{-\left[{∆∆C}_{t}\right]}$), where Δ*C_t_* is the C_t_ value of the gene of interest (GOI) minus the C_t_ value of *GAPDH*; ΔΔ*C_t_* is the particular Δ*C_t_* value minus the Δ*C_t_* value of the calibrator of an assay; and calibrator is the sample with the highest Δ*C_t_* value.

### 2.4. Statistical Analyses

We performed statistical analyses using GraphPad Prism 9 (La Jolla, CA, USA). All results were presented as median values with a range. To evaluate differences between independent cohorts, we used the Mann–Whitney U-test as well as the Kruskal–Wallis test. The correlations of variables were assessed using Spearman rank correlation coefficient. Statistically significant results were considered, as the *p*-value was less than 0.05.

## 3. Results

### 3.1. Aberrant mRNA Expression of BTLA, CD160, SPN, TIM3, VISTA, TIGIT in CLL and Psoriatic Patients Compared to HVs

The expression of *BTLA*, *CD160*, *SPN*, *TIM3*, *VISTA*, *TIGIT* was confirmed in CLL and psoriatic patients.

**Figure 1:**
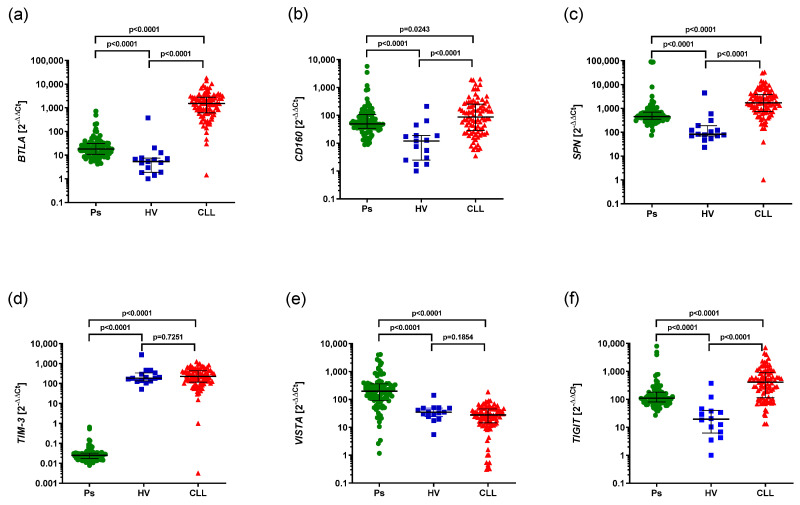
The aberrant expression of *BTLA*, *CD160*, *SPN*, *TIM3*, *VISTA*, *TIGIT* in CLL and psoriatic patients compared to HVs. (**a**) Higher expression of *BTLA* in in CLL patients, as well as Ps patients, compared to HVs (1500 vs. 5.372, *p* < 0.0001), (18.22 vs. 5.372, *p* < 0.0001). Higher expression of *BTLA* in CLL patients than psoriatic patients (1500 vs. 18.22, *p* < 0.0001). (**b**) Higher expression of *CD160* in CLL patients than HVs (86.94 vs. 11.96, *p* < 0.0001). Higher expression of *CD160* in psoriatic patients than HVs (48.92 vs. 11.96, *p* < 0.0001). Higher expression of *CD160* in CLL than Ps patients (86.94 vs. 48.92, *p* = 0.0243). (**c**) Higher expression of *SPN* in CLL patients than HVs (1706 vs. 82.24, *p* < 0.0001). Higher expression of *SPN* in psoriatic patients than HVs (451.8 vs. 82.24, *p* < 0.0001). Higher expression of *SPN* expression in CLL patients than Ps patients (1706 vs. 451.8, *p* < 0.0001). (**d**) Lower expression of *TIM-3* in Ps patients than HVs (0.02485 vs. 183.1, *p* < 0.0001). Higher expression of *TIM-3* in CLL than Ps patients (226.9 vs. 0.02485, *p* < 0.0001). No difference in *TIM-3* expression in CLL patients than HVs (226.9 vs. 183.1, *p* = 0.7251). (**e**) Higher expression of *VISTA* in Ps patients than HVs (196.7 vs. 34.93, *p* < 0.0001). Higher expression of *VISTA* in Ps patients compared to CLL patients (196.7 vs. 27.50, *p* < 0.0001). No difference in *VISTA* expression in CLL patients than HVs (27.50 vs. 34.93, *p* = 0.1854). (**f**) Higher expression of *TIGIT* in CLL patients than Ps patients (409.6 vs. 109.9, *p* < 0.0001). Higher expression of *TIGIT* in CLL patients than HVs (409.6 vs. 19.41, *p* < 0.0001). Higher expression of *TIGIT* in Ps patients than HVs (109.9 vs. 19.41, *p* < 0.0001). The bars spans from Q1 to Q3, and represents the interquartile range (IQR); a line marks the median.

*BTLA* expression was shown to be higher in CLL patients, as well as Ps patients, compared to HVs (1500 vs. 5.372, *p* < 0.0001), (18.22 vs. 5.372, *p* < 0.0001), respectively. Moreover *BTLA* expression was higher in CLL patients compared to psoriatic patients (1500 vs. 18.22, *p* < 0.0001) (Figure 1a). Similarly, *CD160* expression was observed to be higher in CLL patients, as well as Ps patients, compared to HVs (86.94 vs. 11.96, *p* < 0.0001), (48.92 vs. 11.96 *p* < 0.0001), respectively, and *CD160* expression was higher in CLL patients compared to Ps patients (86.94 vs. 48.92, *p* = 0.0243) (Figure 1b). Additionally, *SPN* expression was higher in CLL patients, as well as Ps patients, compared to HVs (1706 vs. 82.24 *p* < 0.0001), (451.8 vs. 82.24 *p* < 0.0001), respectively, and *SPN* expression was higher in CLL patients compared to Ps patients (1706 vs. 451.8 *p* < 0.0001) (Figure 1c).

*TIM-3* expression was significantly lower in Ps patients compared to HVs (0.02485 vs. 183.1; *p* < 0.0001). CLL patients showed significantly higher levels of *TIM-3* expression compared to Ps patients (226.9 vs. 0.02485; *p* < 0.0001). In contrast, there was no statistically significant difference in *TIM-3* expression between patients with CLL and HVs (226.9 vs. 183.1; *p* = 0.7251) (Figure 1d).

*VISTA* expression was found to be higher in Ps patients compared to HVs (196.7 vs. 34.93, *p* < 0.0001) and Ps patients compared to CLL patients (196.7 vs. 27.50, *p* < 0.0001). There were no statistically significant differences in *VISTA* expression in CLL patients compared to HVs (27.50 vs. 34.93, *p* = 0.1854) (Figure 1e).

*TIGIT* expression was shown to be higher in CLL patients compared to Ps patients (409.6 vs. 109.9 *p* < 0.0001), as well as in CLL patients compared to HVs (409.6 vs. 19.41, *p* < 0.0001), and in Ps patients compared to HVs (109.9 vs. 19.41, *p* < 0.0001) (Figure 1f).

### 3.2. Correlations Between Expression of BTLA, CD160, SPN, TIM3, VISTA, as Well as TIGIT in CLL

**Figure 2:**
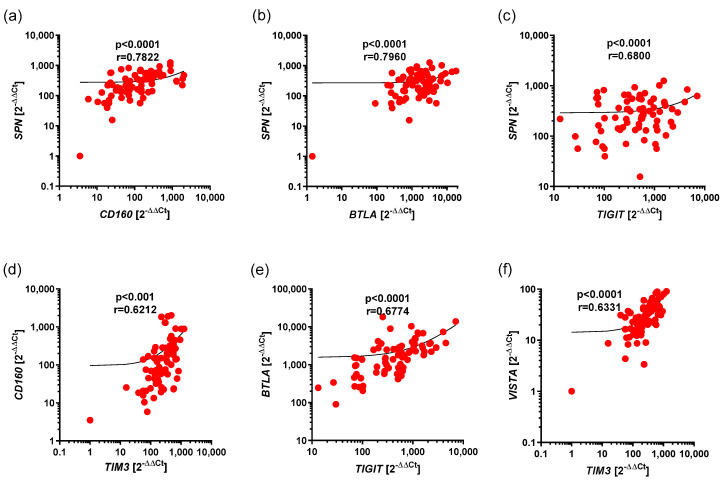
Positive correlations between expression of *BTLA*, *CD160*, *SPN*, *TIM3*, *VISTA*, and *TIGIT* in CLL. The results are presented as the log10 value of 2^−∆∆Ct^, with the regression line marked. (**a**) Strong correlation between the expressions of *SPN* and *CD160* (r = 0.7822, *p* < 0.0001). (**b**) Strong correlation between the expressions of *SPN* and *BTLA* (r = 0.7960, *p* < 0.0001). (**c**) Moderate correlation between the expressions of *SPN* and *TIGIT* (r = 0.6800, *p* < 0.0001). (**d**) Moderate correlation between the expressions of *CD160* and *TIM3* (r = 0.6212, *p* < 0.0001). (**e**) Moderate correlation between the expressions of *BTLA* and *TIGIT* (r = 0.6774, *p* < 0.0001). (**f**) Moderate correlation between the expressions of *TIM3* and *VISTA* (r = 0.6331, *p* < 0.0001).

We showed statistically significant, positive correlations between expressions of the following pairs of genes, including *SPN* and *CD160* (r = 0.7822, *p* < 0.0001), *SPN* and *BTA* (r = 0.7960, *p* < 0.0001), *SPN* and *TIGIT* (r = 0.6800, *p* < 0.0001), *CD160* and *TIM3* (r = 0.6212, *p* < 0.0001), and *BTLA* and *TIGT* (r = 0.6774, *p* < 0.0001), as well as *TIM3* and *VISTA* (r = 0.6331, *p* < 0.0001), in CLL (Figure 2a–f).

The other correlations that have lower impact (r < 0.62) are summarized in Table 3.

### 3.3. Correlations Between the Expression of BTLA, CD160, SPN, TIM3, and VISTA, as Well as TIGIT, in Ps

**Figure 3:**
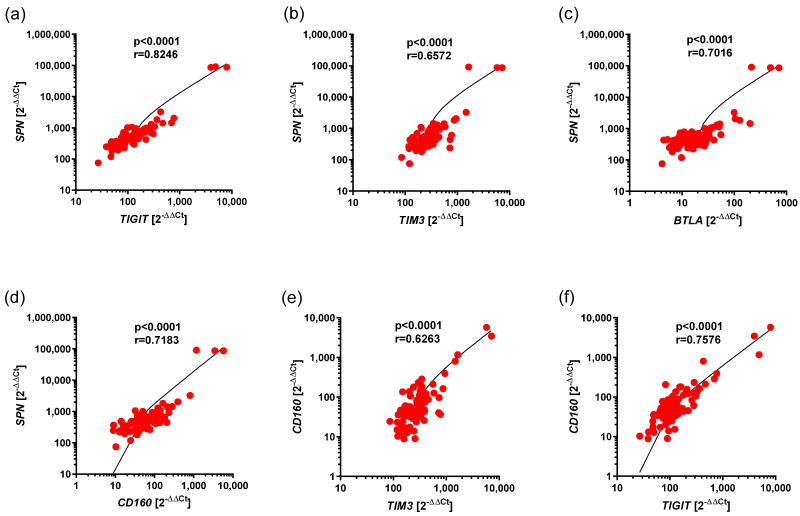
Positive correlations between expression of *BTLA*, *CD160*, *SPN*, *TIM3*, *VISTA*, *TIGIT* in Ps. The results are presented as the log10 value of 2^−∆∆Ct^, with the regression line marked. (**a**) Strong correlation between the expressions of *SPN* and *TIGIT* (r = 0.8246, *p* < 0.0001). (**b**) Moderate correlation between the expressions of *SPN* and *TIM3* (r = 0.6572, *p* < 0.0001). (**c**) Strong correlation between the expressions of *SPN* and *BTLA* (r = 0.7016, *p* < 0.0001). (**d**) Strong correlation between the expressions of *SPN* and *CD160* (r = 0.7183, *p* < 0.0001). (**e**) Moderate correlation between the expressions of *CD160* and *TIM3* (r = 0.6263, *p* < 0.0001). (**f**) Strong correlation between the expressions of *CD160* and *TIGIT* (r = 0.7576, *p* < 0.0001).

We showed statistically significant, positive correlations between expressions of the following pairs of genes, including *SPN* and *TIGT* (r = 0.8246, *p* < 0.0001), *SPN* and *TIM3* (r = 0.672, *p* < 0.0001), *SPN* and *BTLA* (r = 0.7016, *p* < 0.0001), *SPN* and *CD160* (r = 0.7183, *p* < 0.0001), *CD160* and *TIM3* (r = 0.6263, *p* < 0.0001), and *CD160* and *TIGIT* (r = 0.7576, *p* < 0.0001), in Ps (Figure 3a–f).

The other correlations that were lower impact (r < 0.62) are summarized in Table 4.

### 3.4. Associations of the Expression of BTLA, CD160, SPN, TIM3, VISTA, and TIGIT with Prognostic Parameters in CLL

**Figure 4:**
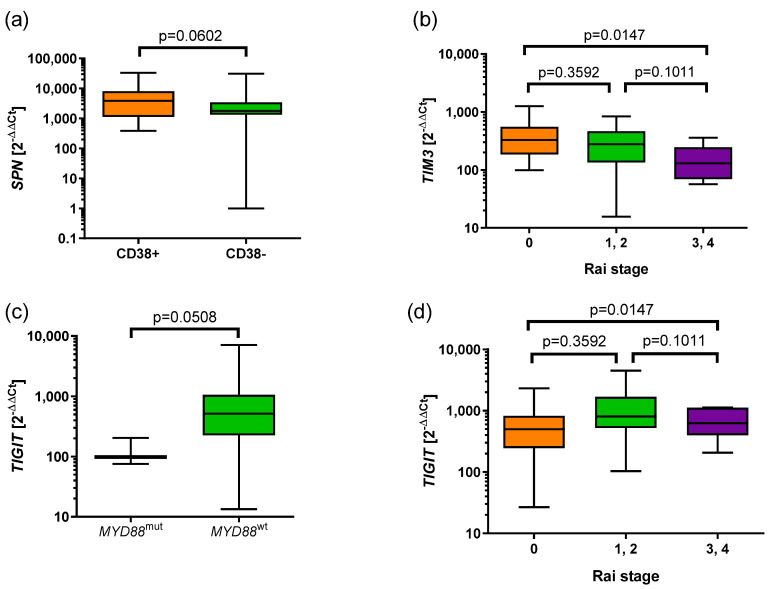
Associations of the expression of *SPN*, *TIM3*, and *TIGIT* with prognostic parameters in CLL. (**a**) Tendency for higher *SPN* expression in CD38+ group compared to CD38− (3864 vs. 1806, *p* = 0.0602). (**b**) Higher expression of *TIM3* in the CLL group with 0 Rai stage compared to stages 3 and 4 (328.1 vs. 130.2, *p* = 0.0186). Tendency for higher expression of *TIM3* was observed in CLL groups with stages 1 and 2 compared to stages 3 and 4 (278.8 vs. 130.2, *p* = 0.0999). (**c**) Tendency for lower *TIGIT* in *MYD88*^mut^ group than *MYD*88^wt^ group (514.2 vs. 98.18). (**d**) Higher expression of *TIGIT* in CLL groups with stages 1 and 2 stage to stage 0, according to Rai classification (806.3 vs. 500.6, *p* = 0.0263), as well as in CLL groups in stages 1, 2, 3, and 4 compared to stage 0 (647.4 vs. 500.6, *p* = 0.0300). The box spans from Q1 to Q3 and represents the interquartile range (IQR); a line inside the box marks the median; whiskers show the smallest and largest values.

To assess the clinical significance of the expression of *BTLA*, *CD160*, *SPN*, *TIM3*, *VISTA*, and *TIGIT* in CLL, we analyzed associations of that expression with prognostic factors including the following: the mutational status of the immunoglobulin heavy-chain variable region *(IGHV)*, *MYD88*, *TP53*, and *NOTCH1*, and the expression of the zeta chain of T-cell receptor-associated protein kinase 70 (ZAP-70), CD38, and lactate dehydrogenase (LDH) activity and β_2_microglobulin level. Moreover, we analyzed differences in those expressions with the clinical stage of CLL, according to the Rai stage. We observed a tendency for higher *SPN* expression in the CD38+ group compared to CD38− (3864 vs. 1806, *p* = 0.0602) (Figure 4a).

The differences in the *TIM3* expression in referring to the stage of disease, according to the Rai classification, were shown. Higher expression of *TIM3* was observed in the CLL group with stage 0 compared to stages 3 and 4 (328.1 vs. 130.2, *p* = 0.0186). Moreover, a tendency for higher expression of *TIM3* was observed in CLL groups with stages 1 and 2 compared to stages 3 and 4 (278.8 vs. 130.2, *p* = 0.0999) (Figure 4b).

We showed a tendency for lower *TIGIT* in the *MYD88*^mut^ group compared to the *MYD*88^wt^ group (514.2 vs. 98.18, *p* = 0.0508) (Figure 4c). Higher expression of *TIGIT* was observed in CLL groups with stages 1 and 2, compared to stage 0, according to the Rai classification (806.3 vs. 500.6, *p* = 0.0263), as well as in CLL groups in stages 1,2,3 and 4, compared to stage 0 (647.4 vs. 500.6, *p* = 0.0300) (Figure 4d).

**Figure 5:**
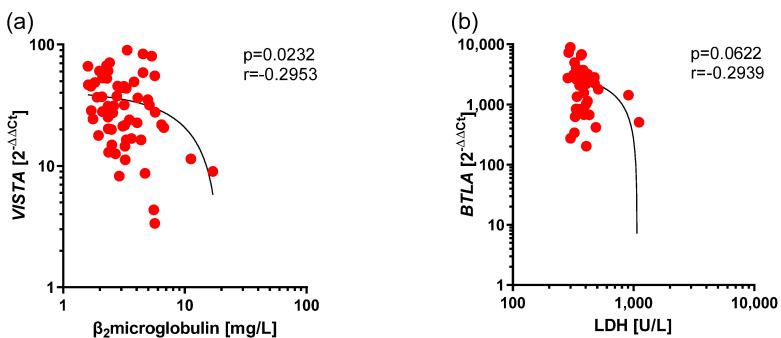
Negative correlations between expression of *VISTA* and *BTLA* and prognostic factors in CLL. The results are presented as the log10 value of 2^−∆∆Ct^ with the regression line marked. (**a**) Low correlation between *VISTA* expression and β_2_microglobulin level (r = −0.2953, *p* = 0.0232). (**b**) Low correlation between *BTLA* expression and level of LDH (r = −0.2939, *p* = 0.0622).

Low, negative correlation between *VISTA* expression and β_2_microglobulin level (r = −0.2953, *p* = 0.0232) (Figure 5a) and low, negative correlation between *BTLA* expression and level of LDH (r = −0.2939, *p* = 0.0622) (Figure 5b) were provided.

No statistically significant analyses with the CLL prognostic factors are summarized in Supplementary Table S1a,b.

### 3.5. Associations of the Expression of BTLA, CD160, SPN, TIM3, VISTA, and TIGIT and Clinical Parameters in Ps

**Figure 6:**
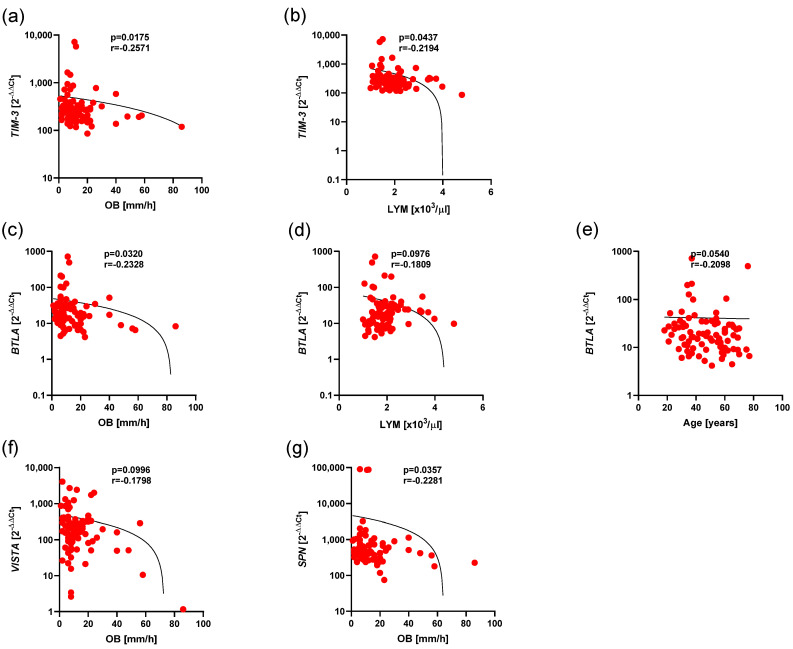
Negative correlations between expression of *BTLA*, *SPN*, *VISTA*, *TIM3* and clinical parameters in Ps. The results are presented as the log10 value of 2^−∆∆Ct^, with the regression line marked. (**a**) Low correlation between *TIM3* expression and OB level (r = −0.2571, *p* = 0.0175). (**b**) Low correlation between *TIM3* expression and leukocyte level (r = −0.2194, *p* = 0.0437). (**c**) Low correlation between *BTLA* expression and OB level (r = −0.2328, *p* = 0.0320). (**d**) Low correlation between *BTLA* expression and lymphocyte level (r = −0.1809, *p* = 0.0976). (**e**) Low correlation between *BTLA* expression and age level (r = −0.2098, *p* = 0.0540). (**f**) Low correlation between *VISTA* expression and OB level (r = −0.1798, *p* = 0.0996). (**g**) Low correlation between *SPN* expression and OB level (r = −0.2281, *p* = 0.0357).

To assess the clinical significance of the expression of *BTLA*, *CD160*, *SPN*, *TIM3*, *VISTA*, and *TIGIT* in Ps, we analyzed associations of those expressions with prognostic factors, including gender, type of Ps, psoriatic arthritis activity, clinical course, age and duration, and also PASI, WBC, neutrophils, lymphocytes, CRP and OB level. We showed a low, negative correlation between *TIM3* expression and OB level (r = −0.2571, *p* = 0.0175) (Figure 6a), and a low, negative correlation between *TIM3* expression and lymphocyte level (r = −0.2194, *p* = 0.0437) (Figure 6b).

Additionally we showed a low, negative correlation between *BTLA* expression with OB level (r = −0.2328, *p* = 0.0320) (Figure 6c); also, a low, negative correlation between *BTLA* expression and lymphocytes level (r = −0.1809, *p* = 0.0976) (Figure 6d); and a low, negative correlation between *BTLA* expression and age level (r = −0.2098, *p* = 0.0540) (Figure 6e). In addition, we observed a low, negative correlation between *VISTA* expression and OB level (r = −0.1798, *p* = 0.0996) (Figure 6f), and a low, negative correlation between *SPN* expression and OB level (r = −0.2281, *p* = 0.0357) (Figure 6g).

**Figure 7:**
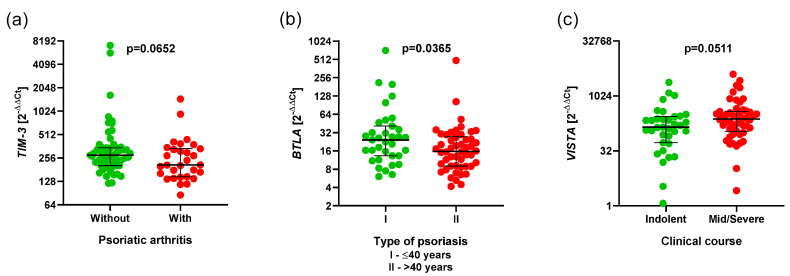
The aberrant expressions of *BTLA*, *TIM3*, *VISTA* and clinical parameters in Ps. The results are presented as the log2 value of 2^−∆∆Ct^, with the regression line marked. (**a**) Higher expression of *TIM3* in the group without psoriatic arthritis compared to the group with psoriatic arthritis (276.1 vs. 209.1, *p* = 0.0652). (**b**) Higher expression of *BTLA* in type I Ps group compared to type II Ps group (24.19 vs. 15.70 *p* = 0.0365). (**c**) Lower expression of *VISTA* in mild clinical course than with severe clinical course (143.1 vs. 239.3, *p* = 0.0511). The bars spans from Q1 to Q3 and represents the interquartile range (IQR); a line marks the median.

A tendency for higher *TIM3* expression in the group without psoriatic arthritis compared to the group with psoriatic arthritis (276.1 vs. 209.1 *p* = 0.0652) (Figure 7a), and, also, higher *BTLA* expression in the type I Ps group compared to the type II Ps group, were observed (24.19 vs. 15.70 *p* = 0.0365) (Figure 7b). Additionally, we observed a tendency for lower *VISTA* expression with a mild clinical course than with a severe clinical course (143.1 vs. 239.3 *p* = 0.0511) (Figure 7c).

No statistically significant analyses with the Ps prognostic factors are summarized in Supplementary Table S2a,b.

## 4. Discussion

In our research, we provide a collective screening of the immune-regulatory genes expression, including *BTLA*, *CD160*, *SPN*, *TIM-3*, *VISTA* and *TIGIT* on the mRNA level, as well as a comparison for two different diseases, such as CLL and Ps. We referred the mRNA expression to recognized prognostic markers, as well as clinical characteristics in CLL and in Ps. We showed aberrant expression of the following genes in those two diseases.

We showed that *BTLA* expression was higher in CLL patients compared to Ps patients and higher in Ps and CLL patients compared to HVs. Moreover we observed a negative correlation between *BTLA* expression and the level of LDH. The other associations of mRNA expression of *BTLA* with known prognostic factors in CLL were not statistically significant. Notably, BTLA research has been focused mainly on T cells. There is little research on its function in B cells [23]. It is known that BTLA increases the expression of T regulatory cells, and it provides a negative regulatory effect on Th17, as well as Th1 cell immune responses. In addition to inhibiting lymphocyte activation through TCR-mediated signal transduction and inhibiting cytokine (IL-2, IL-4 and IL-10) secretion, BTLA also crosslinks with herpesvirus entry mediator (HVEM) on Treg cells, enabling its immunosuppressive effects. In addition, BTLA inhibits production of immunoglobulins G (IgG) by suppressing IL-21 secretion by follicular helper T cells (Tfhs) and plays an important role in immunomodulation in body fluids. Interestingly, BTLA inhibits the proliferation of T lymphocytes γδ and secretion of IL-17, TNF-α. So far, one paper has investigated BTLA expression in Ps, with a study group of 25 patients and a control group of 25 healthy people. It was shown that the expression of the *BTLA* gene was significantly lower in Ps [24]. The divergent research results in our study and in the study by Youseff R. et al. [24] may result from the small control group and the study group, which in this case consisted of 25 people. An increased expression of BTLA and its ligand HVEM has been shown in cancer and, particularly, in B-cell lymphoproliferations, including CLL. In CLL, high expression of HVEM was also demonstrated, which may indicate that cells engage in BTLA/HVEM interaction to inhibit T cell responses and are engaged in immunosuppression of this population of immune cells. Moreover, it was shown that BLTA expression provides a prognostic role in CLL [25,26]. Interestingly, previous studies showed that BTLA is a receptor which inhibits the B-cell receptor (BCR) signaling pathway that is crucial for B-cell activation. BTLA inhibits the strength of signaling through BCR by recruiting and phosphorylating the protein tyrosine kinase Syk and down-regulating B cell linker proteins, and phospholipase E2, as well as NF-κB [27]. Ware et al. [28] indicated that HVEM/BTLA signaling can suppress B-cell proliferation and CPG oligonucleotide-mediated cytokine secretion and increase stimulatory molecules on their surface. However, IL-8 and macrophage inflammatory protein 1β (MIP1β) secretion are not affected, indicating that BTLA may partially inhibit B-cell function. However, studies also indicated that BTLA expression in B cells is reduced in elderly patients, leading to less response to the H1N1 virus and an inability to produce IgG antibodies, resulting in lower vaccine responses [29]. Thereby, this implies that BTLA may have dual regulatory roles in certain cases, depending on the context [30,31,32]. Remarkably, it was shown that there are drugs that can selectively activate BTLA that might achieve long-term disease remission of immune-mediated inflammatory diseases, including ANB032, a BTLA agonist antibody. This antibody provided promising results in modulation of the immune response by inhibiting T cell proliferation and cytokine production, which are crucial in the pathogenesis of diseases like atopic dermatitis [33]. A preclinical study showed that ANB032 modulated the cell function of dendritic cells, induced Treg, and restored immune balance [34]. The first phase of clinical trials also demonstrated favorable safety and pharmacokinetics, with sustained BTLA receptor occupancy and reduced BTLA expression on T and B cells for over 30 days, suggesting its potential for long-term disease management [34].

In our study, *CD160* expression was observed to be higher in Ps patients compared to HVs. This may be related to the severe skin condition in our patients. Our publication is most likely the first to discuss *CD160* expression in Ps patients. We showed higher mRNA expression of *CD160* in CLL compared to Ps and HVs. However, no associations with clinical factors, including prognostic markers, are observed. In the literature, there is still limited data on the mRNA expression of *CD160* in CLL. The other studies confirmed CD160 is expressed on the protein level on most NK cells as an immunoglobulin-like activating receptor. CD160 is expressed on some CD8+ T cells, but is not expressed on healthy B cells [35]. In CLL, CD160 has been shown to mediate PI3K-dependent regulation of cell activation, and positive regulation (upregulation) of Bcl-2 and Bcl-XL proteins. In addition, CD160 improved cell survival in vitro and cell secretion of cytokines. The limited expression of CD160 in the B cell line as a surface marker on CLL, but not on normal B cells, makes it an ideal marker used to detect minimal residual disease (MRD) in CLL [36]. Signal transduction through CD160 mediates PI3K-dependent signals for cell survival and growth in CLL [2,35]. Protein expression of ligands for CD160 has been demonstrated, both on leukemic cells and on other cells in the lymphoid microenvironment. These ligands include MHC class I molecules, CD1d and HLA-G. It appears that CD160 interactions with its ligands may play an important role in the pathophysiology of malignant B cells through autocrine, paracrine and/or stromal cell interactions, offering new targets for therapeutic strategies [37].

Moreover, we showed increased *SPN* mRNA expression levels in Ps patients compared to healthy volunteers. This may be due to the fact that the Ps in our study were active, and that all patients were without general treatment at the time of the study, and had severe Ps. Currently, there is no literature regarding the expression of *SPN* at the mRNA level in Ps. Recruitment of T cells to the skin is a central feature of many acute and chronic inflammatory skin conditions, including eczema, Ps, vitiligo, and alopecia areata. A subpopulation of memory effector T lymphocytes participates in the immune response in the skin, which can be identified by the presence of lymphocytes with positive expression of the antigen associated with cutaneous T lymphocytes CLA+ (cutaneous lymphocyte-associated antigen CLA [2,38]. It has been shown that CD43 is a ligand for P-selectin-1 (PSGL-1) present on CLA+ T cells [39]. In CLL, we observed higher expression of *SPN* (CD43) compared to HVs, as well as compared to Ps. Assessment of protein expression at the CD43 protein level has been shown to enable differential diagnosis between CLL and other malignancies, with proliferation of mature B lymphocytes [40,41]. Moreover, CD43 expression was assessed by flow cytometry as applicable to protocols for assessing minimal residual disease in CLL patients [41]. However, there are no reports regarding *SPN* expression at the mRNA level in CLL [42]. We observed a tendency to higher *SPN* expression in the CD38+ group compared to CD38−. However, there were no associations with the other known prognostic factors.

Various inhibitory receptors, known as immune checkpoints, are engaged in regulating the activity of T cells, as well as NK cells. Many cancer cells use these molecules to escape the anti-tumor immune response. Abnormal regulation of these receptors has been identified in several hematologic malignancies [43]. Among the inhibitory molecules, Tim-3 plays a significant role in immune tolerance through negative regulation of pro-inflammatory signaling [44]. Our study showed reduced *TIM-3* levels in Ps patients compared to healthy volunteers. Tim-3 is a regulatory protein that has different effects, depending on the context, and may have a positive or negative impact on the immune response. It has been proven that Tim-3 expression is reduced in autoimmune diseases such as rheumatoid arthritis and Ps, which is confirmed by our research results [44]. Interestingly, our paper also provided higher expression of *TIM-3* in CLL than Ps patients and no difference in *TIM-3* expression in CLL patients compared to HVs. However, we showed higher expression of *TIM3* in the CLL group with stage 0 compared to stages 3 and 4, according to the Rai stage classification, and a tendency for higher expression of *TIM3* was observed in CLL groups with stages 1 and 2 compared to stages 3 and 4, which indicates higher expression of *TIM-3* in the earlier clinical stage of disease and suggests its possible diagnostic value in CLL. The other studies proved that immune cells, especially T cells of CLL patients, show higher expression of various inhibitory receptors, such as Tim-3, as well as PD-1 and CTLA4, which constitute immune checkpoints eventually leading to T cell depletion [30,45,46,47]. It was shown that CLL patients with a more progressive type of disease have a higher percentage of PD-1-expressing T cells in the peripheral blood compared to healthy controls at the protein and mRNA levels [47]. Moreover, it was shown that during CLL progression, significantly higher Tim-3 and PD-1 expression was observed on both CD8+ and CD4+ T cells, accompanied by significant functional defects in these cells [48]. Tim-3 expression was significantly higher in NK cells of CLL patients compared to HVs. Moreover, NK cells from CLL patients showed lower expression of the NKp30 activating receptor compared to HVs. Abnormalities in the regulation of Tim-3 and NKp30 receptor expression indicated the exhaustion state of NK cells in CLL [20]. Tim-3 expression on the protein level has been shown in other leukemias [48]. Increased expression of Tim-3 has been reported on leukemic stem cells (LSCs), and not on healthy HSCs in MDS [48,49].Therefore, TIM3 could represent a promising marker for detecting malignant clone cells in MDS, as well as being a candidate for targeted therapy [49]. Overexpression of TIM-3 on exhausted CD4+ and CD8+ T cells and leukemic cells in patients with chronic lymphocytic leukemia (CML), acute lymphoblastic leukemia (ALL), and CLL, might be a prognostic factor for poor therapeutic response and relapse in patients. Significantly, several TIM-3 inhibitors are being checked in clinical trials for leukemias, especially in MDS and AML [48,50].

Our analyses showed increased mRNA expression of *VISTA* and *TIGIT* in Ps patients compared to healthy volunteers. Previous work has proven that the expression of VISTA and TIGIT may vary, depending on the clinical context. The work of Li et al. [21] showed that in an imiquimod (IMQ)-induced mouse model of Ps, Vsir−/−mice developed more severe psoriatic inflammation compared to WT mice. VISTA regulated IL-17 production by both γδ T cells and CD4+ Th17 cells. Expression of VISTA on dendritic cells inhibited IMQ-induced TLR7 signaling and IL-23 production [21]. The expression of VISTA and TIGIT can be compared to the action of the PD-1 protein. Each of these genes can act in a dual way. The lability of expression may be caused by many factors that are difficult to identify; for example, the severity of the disease. The research conducted so far shows that the genes tested will not be useful as markers of inflammation [51]. Moreover, under inflammatory conditions, VISTA expression on different types of immune cells can be altered. It was shown that on human CD14+ monocytes, surface expression of VISTA can be positively upregulated after stimulation of certain TLR receptors, such as TLR3, TLR5, and the cytokines IL-10, IFN-γ, as well as after HIV infection. At the transcriptional level, VISTA, as well as PD-L1 and PD-1, constitute a direct target for the tumor suppressor p53. Upregulated transcription occurs following forced expression of p53 or p53-induced genotoxic stress [21]. Our report showed higher expression of *VISTA* in Ps patients compared to CLL patients, and no difference in *VISTA* expression in CLL patients compared to HVs. We observed no associations of *VISTA* expression with clinical characteristics, only a low, negative correlation between *VISTA* expression and β_2_microglobulin level, as we have provided. There are no more reports on the expression and role of VISTA in CLL. However, VISTA is highly expressed on bone marrow-derived suppressor cells (MDSCs) of acute myeloid leukemia (AML) patients [52]. Both the intensity and percentage of VISTA expression on MDSCs are significantly higher in newly diagnosed AML than in HVs. Notably, exclusion of VISTA by specific siRNA significantly reduced MDSC-dependent inhibition of CD8 T-cell activity in AML, suggesting a suppressive effect of VISTA on the anti-leukemic T-cell response. A strong positive association was observed between VISTA expression by MDSCs and PD-1 expression by T cells in AML.

In our study, we provided higher expression of *TIGIT* in CLL patients than in Ps patients, as well as higher expression of *TIGIT* in CLL patients than in HVs. Additionally, higher expression of *TIGIT* in CLL groups with stages 1 and 2 compared to stage 0, according to the Rai classification, as well as in CLL groups in the 1, 2, 3 and 4 stages compared to stage 0 was observed, which might suggest the more important impact in the more developed disease. However, we also showed a tendency for lower *TIGIT* in the *MYD88*^mut^ group than in *MYD*88^wt^. The other studies [53,54] demonstrated that TIGIT expression is significantly increased in CD4+ T-cell in CLL patients and is positively correlated with PD-1 expression in the same cells, on the protein level. The percentage of T lymphocytes with co-expression of TIGIT+ and CD4+ is higher in CLL patients with a worse prognosis, determined by advanced disease stage, unmutated *IGHV* genes, or unfavorable cytogenetics. Functionally, T cells with the co-expression of TIGIT+ and CD4+ show an enhanced ability to maintain leukemic cell survival in vitro in co-cultures. Moreover blocking TIGIT interactions with recombinant TIGIT-Fc molecules reduces CLL cell viability and interferes with the secretion of anti-apoptotic cytokines by CD4+ T cells [55]. As with other immunomodulatory molecules, therapeutic antibodies targeting TIGIT have been developed and have recently entered clinical trials, limited to solid metastatic tumors. No trials are currently underway for CLL [4,56].

Additionally, we provided statistically significant correlations between expressions of the following genes with the most statistical significance for paired genes, including *SPN* and *CD160*, *SPN* and *BTLA*, *SPN* and *TIGIT*, *CD160* and *TIM3*, *BTLA* and *TIGT* and *TIM3* and *VISTA* in CLL, and for *SPN* and *TIGT*, *SPN* and *TIM3*, *SPN* and *BTLA*, *SPN* and *CD160*, *CD160* and *TIM3*, *CD160* and *TIGIT* in Ps. Those correlation patterns of mRNA transcripts may suggest similar regulation in CLL and Ps patients. Moreover, correlations between gene expressions of TIM3, *BTLA*, *VISTA* and *SPN* with OB might suggest their possible negative immunoregulatory impact on nonspecific immune reactions.

To sum up, we have characterized the expressions of *BTLA*, *CD160*, *SPN*, *TIM-3*, *TIGIT* and *VISTA* in CLL and Ps compared to HVs. In Ps, all the studied gene expressions, except *TIM-3*, were higher than in HVs and all the studied gene expressions, except *VISTA*, were lower than in CLL. However, the expression of *TIM-3*, a checkpoint inhibitor, was higher in stage 0 of CLL and it was lower in more advanced stages of the disease, suggesting its possible diagnostic value in CLL. Moreover, expression of *VISTA* was higher in Ps than in HVs, as well as in CLL. Of particular note, *BTLA*, *CD160*, *SPN* and *TIGIT* were over-expressed in CLL and Ps compared to HVs, suggesting their involvement in immune suppression in both diseases. Significant correlations between gene expressions of *SPN* and *BTLA*, *SPN* and *TIGIT*, *CD160* and *TIM-3* were observed, indicating a potential shared regulatory mechanism for immune responses in both diseases; this suggests their bidirectional regulatory role on the functioning of immune system cells, depending on the context of inflammatory or neoplastic diseases.

Due to the substantial role of the studied genes in modulating immune response, they may appear to be a new target for therapeutic strategies.

## 5. Conclusions

Overall, the findings highlight disease-specific patterns of immune checkpoint molecule expression, and suggest that the deregulation of immune inhibitory pathways could be crucial in both CLL and Ps pathophysiology. Although Ps is a classical autoimmune disease characterized by excessive immune activation, and CLL is a hematological malignancy marked by immune suppression and escape, interestingly, we observed some overlapping patterns of immune checkpoint dysregulation. This points to a broader dysfunction of immune regulation in both conditions. Notably, CLL might be accompanied by autoimmune phenomena such as autoimmune hemolytic anemia or immune thrombocytopenia, suggesting that elements of autoimmune imbalance exist even in the context of malignancy. These similarities may reflect common underlying mechanisms of immune exhaustion, dysregulated T-cell responses, or chronic immune stimulation. Furthermore, the aberrant expression of *BTLA*, *TIGIT*, and *TIM-3* in both diseases highlights potential therapeutic targets for restoring immune balance. It is tempting to speculate that targeting specific immune checkpoints might not only improve anti-tumor immunity in CLL, but also modulate autoimmunity-related manifestations observed in a subset of CLL patients. Future studies exploring the functional consequences of these molecular alterations are warranted to better understand their role in disease progression and therapeutic resistance.

## Figures and Tables

**Table 1:** Clinical characteristics of CLL patients.

Characteristic	CLL (*n* = 74)
**Sex**	
Male	46
Female	28
**Age (years)**	
Median	66
Range	48–84
**Rai Stage**	
0	26
I–II	34
III–IV	14
**ZAP-70 (cut-off 20%)**	
Positive	24
Negative	34
NA	16
**CD38 (cut-off 30%)**	
Positive	22
Negative	38
NA	14
***IGHV***	
Mutated	32
Unmutated	39
NA	3

Shortcuts: *IGHV*-immunoglobulin heavy-chain variable region, ZAP-70-zeta chain of T-cell receptor-associated protein kinase 70, CD38-cluster of differentiation 38

**Table 2:** Clinical characteristics of Ps patients.

Characteristic	Ps (*n* = 85)
**Sex**	
Male	71
Female	14
**Age (years)**	
Median	47
Range	18–77
**Type**	
I age ≤ 40	35
II age > 40	50
**Articular Ps**	
With	30
Without	55
**Duration**	
Median	16
Range	55
**PASI**	
Median	12.1
Range	49.4
**Course**	
Mild	35
Severe	50
**WBC**	
Median	6.53
Range	3.54–13.42
**Neutrophils**	
Median	3.61
Range	1.4–10.96
**Lymphocytes**	
Median	1.83
Range	1–4.79
**CRP**	
Median	1.6
Range	0.8–57.3
**OB**	
Median	9
Range	1–86

Shortcuts: PASI—psoriasis area severity index, CRP—human C-reactive protein, WBC—white blood cells.

**Table 3:** Correlations between gene expressions in CLL.

Pairs of Genes	r	Statistical Significance (*p*)
*TIGIT* and *TIM3*	0.2522	0.068
*TIM3* and *BTLA*	0.4003	<0.001
*CD160* and *VISTA*	0.3861	<0.001
*CD160* and *BTLA*	0.5504	<0.0001
*CD160* and *SPN*	0.5821	<0.0001

**Table 4:** Correlations between gene expressions in Ps.

Pairs of Genes	r	Statistical Significance (*p*)
*TIGIT* and *TIM3*	0.5951	<0.0001
*TIM3* and *BTLA*	0.5312	<0.0001
*TIGIT* and *BTLA*	0.6012	<0.0001
*BTLA* and *CD160*	0.4773	<0.0001

## Data Availability

The data supporting the findings of our study are available on request from the corresponding author.

## Supplementary Materials

The following supporting information can be downloaded at: https://www.mdpi.com/article/10.3390/cancers17132116/s1, Table S1a. Associations of the expression of *BTLA*, *CD160*, *SPN*, *TIM3*, *VISTA*, *TIGIT* with prognostic parameters in CLL., Table S1b. Correlations between expression of *BTLA*, *CD160*, *SPN*, *TIM3*, *VISTA*, *TIGIT* with prognostic parameters in CLL. Table S2a. Associations of the expression of *BTLA*, *CD160*, *SPN*, *TIM3*, *VISTA*, *TIGIT* and clinical parameters in Ps. Table S2b. Correlations between associations of the expression of *BTLA*, *CD160*, *SPN*, *TIM3*, *VISTA*, *TIGIT* and clinical parameters in Ps.
