# Supplementary material for: Aberrant Expression of BTLA, CD160, SPN, TIM-3, VISTA and TIGIT in Chronic Lymphocytic Leukemia and Psoriasis Patients Compared to Healthy Volunteers
Source: Cancers (Basel). 2025 Jun 24;17(13):2116. doi: 10.3390/cancers17132116 (PMC12249134; doi:10.3390/cancers17132116)
Supplement: Supplementary file 1 [file cancers-17-02116-s001.zip › cancers-3700479-supplementary.pdf]

Table S1a. Associations of the expression of *BTLA*, *CD160*, *SPN*, *TIM3*, *VISTA*, *TIGIT* with prognostic parameters in CLL.

Gene and parameter	p	Gene and parameter	p
TIM3 and ZAP-70	0.7702	*CD160* and ZAP-70	0.2727
TIM3 and CD38	0.9328	*CD160* and CD38	0.2991
TIM3 and TP53	0.6464	*CD160* and TP53	0.5756
TIM3 and *NOTCH1* ARMS	0.1815	*CD160* and *NOTCH1* ARMS	0.3976
TIM3 and *NOTCH1* ddPCR	0.3033	*CD160* and *NOTCH1* ddPC	0.1189
TIM3 and *IGHV*	0.9677	*CD160* and *IGHV*	0.5450
TIM3 and *MYD88*	0.2962	*CD160* and *MYD88*	0.1524
TIM3 and sex	0.9157	*CD160* and sex	0.7172
VISTA and ZAP-70	0.6940	*CD160* and RAI	0.6285
VISTA and CD38	0.9820	*BTLA* and ZAP-70	0.5320
VISTA and *TP53*	0.1205	*BTLA* and CD38	0.2991
VISTA and *NOTCH1* ARMS	0.8823	*BTLA* and TP53	0.1123
VISTA and *NOTCH1* ddPCR	0.1047	*BTLA* and *NOTCH1* ARMS	0.1494
VISTA and *IGHV*	0.4489	*BTLA* and *NOTCH1* ddPCR	0.4640
VISTA and *MYD88*	0.2175	*BTLA* and *IGHV*	0.9639
VISTA and sex	0.9258	*BTLA* and *MYD88*	0.8694
VISTA and RAI	0.2617	*BTLA* and sex	0.8480
TIGIT and ZAP-70	0.6989	*BTLA* and RAI	0.5500
TIGIT and CD38	0.4206	*SPN* and ZAP-70	0.9811
TIGIT and *TP53*	0.4164	*SPN* and TP53	0.2553
TIGIT and *NOTCH1* ARMS	0.7612	*SPN* and *NOTCH1* ARMS	0.3888
TIGIT and *NOTCH1* ddPCR	0.6008	*SPN* and *NOTCH1* ddPCR	0.1331
TIGIT and *IGHV*	0.5990	*SPN* and *IGHV*	0.1413
TIGIT and sex	0.8892	*SPN* and *MYD88*	0.7705
SPN and RAI	0.5453	*SPN* and sex	0.9463

list of abbreviations: *NOTCH1 ARMS* – mutation of *NOTCH1* assessed by amplification-refractory mutation system PCR (ARMS PCR), *NOTCH1* ddPCR- mutataion of *NOTCH1* assessed by droplet digital PCR (ddPCR)

Table S1b. Correlations between expression of *BTLA*, *CD160*, *SPN*, *TIM3*, *VISTA*, *TIGIT* with prognostic parameters in CLL.

Gene and parameter	r	p
TIM3 and age	0.1826	0.2244
TIM3 and β2microglobulin	-0.1673	0.2093
TIM3 and LDH	0.1854	0.2210
TIGIT and age	-0.06279	0.6820
TIGIT and β2microglobulin	0.09854	0.4578
TIGIT and LDH	0.02718	0.8660
CD160 and age	0.2384	0.1147
CD160 and β2microglobulin	0.05996	0.6548
CD160 and LDH	0.09289	0.5686
CD160 and WBC	-0.01031	0.9325
BTLA and age	-0.03384	0.8233

BTLA and β2microglobulin	0.03931	0.7676
SPN and age	0.1692	0.2666
SPN and β2microglobulin	0.0930	0.4875
SPN and LDH	0.02984	0.8550
SPN and WBC	0.1329	0.2728

Table S2a. Associations of the expression of *BTLA*, *CD160*, *SPN*, *TIM3*, *VISTA*, *TIGIT* and clinical parameters in Ps.

Gene and parameter	p
TIM3 and sex	0.7178
TIM3 and type	0.1970
TIM3 and course	0.3601
VISTA and sex	0.1137
VISTA and type	0.9395
VISTA and articular psorasis	0.7933
BTLA and sex	0.5980
BTLA and articular psorasis	0.8939
BTLA and course	0.9964
TIGIT and sex	0.7716
TIGIT and type	0.8338
TIGIT and articular psorasis	0.6034
TIGIT and course	0.6585
CD160 and sex	0.5107
CD160 and type	0.6456
CD160 and articular psorasis	0.3366
CD160 and course	0.8758
SPN and sex	0.9010
SPN and type	0.4139
SPN and articular psorasis	0.8217
SPN and course	0.3648

Table S2b. Correlations between associations of the expression of *BTLA*, *CD160*, *SPN*, *TIM3*, *VISTA*, *TIGIT* and clinical parameters in Ps.

Gene and parameter	r	p
TIM3 and age	-0.1071	0.3293
TIM3 and duration	-0.0967	0.3787
TIM3 and PASI	0.1256	0.2521
TIM3 and WBC	-0.1456	0.1827
TIM3 and neutrophils	-0.07733	0.4818
TIM3 and CRP	0.05661	0.6068
VISTA with articular psorasis	-0.03728	0.7933
VISTA and age	0.04213	0.7018
VISTA and duration	-0.03728	0.7348

VISTA and PASI	0.1325	0.2266
VISTA and WBC	0.1035	0.3457
VISTA and neutrophils	0.03481	0.7518
VISTA and lymphocytes	0.1772	0.1046
VISTA and CRP	-0.063227	0.5651
BTLA and duration	-0.00726	0.9474
BTLA and PASI	0.01592	0.8850
BTLA and WBC	-0.1334	0.2237
BTLA and neutrophils	-0.1701	0.1197
BTLA and CRP	-0.1058	0.3354
TIGIT and age	0.0326	0.7671
TIGIT and duration	0.1351	0.2177
TIGIT and PASI	0.04231	0.7006
TIGIT and WBC	-0.1184	0.2804
TIGIT and neutrophils	-0.1361	0.2142
TIGIT and lymphocytes	-0.00685	0.9504
TIGIT and CRP	0.09427	0.3908
TIGIT and OB	-0.1636	0.1346
CD160 and age	-0.07158	0.5150
CD160 and duration	-0.04773	0.6645
CD160 and PASI	0.04789	0.6634
CD160 and WBC	-0.08310	0.4496
CD160 and neutrophils	-0.1426	0.1929
CD160 and lymphocytes	0.1051	0.3385
CD160 and CRP	-0.01685	0.8784
CD160 and OB	-0.1534	0.1611
SPN and age	-0.08449	0.4420
SPN and duration	0.05957	0.5881
SPN and PASI	0.1469	0.1799
SPN and WBC	-0.1292	0.2388
SPN and neutrophils	-0.1015	0.3553
SPN and lymphocytes	-0.05792	0.5985
SPN and CRP	-0.02723	0.8046
